# When expressions make impressions—Nurses’ narratives about meeting severely ill patients in home nursing care: A phenomenological-hermeneutic approach to understanding

**DOI:** 10.3402/qhw.v8i0.21880

**Published:** 2013-10-17

**Authors:** Siri Andreassen Devik, Ingela Enmarker, Ove Hellzen

**Affiliations:** 1 Centre of Care Research, Steinkjer, Mid-Norway; 2 Department of Nursing, Mid-Sweden University, Sundsvall, Mid-Sweden; 3 Department of Health Sciences, Nord-Trøndelag University College, Namsos, Norway

**Keywords:** Nurse–patient interactions, emotional dimension, touch, home nursing care, palliative care, professional identity, phenomenological-hermeneutic approach

## Abstract

Registered nurses (RNs) working in homecare encounter severely ill and palliative patients whose expressions may cause ethical challenges and influence their daily work. The aim of this qualitative study was to illuminate and interpret the meaning of nurses’ lived experiences when meeting these patients. Narrative interviews were conducted with 10 RNs working in home nursing care. These interviews were audiotaped and transcribed verbatim to a text and interpreted by a phenomenological-hermeneutic method inspired by Ricoeur. The meaning of the RNs’ lived experience of patients’ expressions was formulated into four themes. The first theme, *Being open for the presence of the Other*, includes two subthemes: “Sensing vulnerability” and “Empathizing with.” The second theme, *Being satisfied*, entails the subthemes, “Feeling exceptional” and “Being trusted.” The third theme, *Being frustrated*, contains the subthemes, “Being disappointed” and “Being angry.” The fourth and final theme, *Being ambivalent*, includes one subtheme: “Being generous or reserved.” Patients’ expressions that make impressions on nurses create emotional waves. Expressions leave impressions that call upon the nurse, and confront her with taking the risk of letting intuition and pre-reflexive feelings gain entry to her care. Allowing for the Other's presence is seen as a precondition, which means facing humanity and sensing a vulnerability in herself as well as in the Other. Understanding and balancing this emotional dimension in care seems to cause confusion and distress within the nurses. Realizing how their feelings may lead to either generosity or aloofness towards the patient is upsetting. Our interpretation suggests that these impressions echo confusion according to the role of being a professional nurse. There is a need to pay more attention to how the emotional dimension in care is understood and impacts the way nurses perform their professional role.

## Introduction

In recent decades, an increasing number of severely ill people have chosen to end their lives in their own homes. To end one's life in dignity at home often requires specialized nursing care (Helme, 2003). In palliative care, the caring relationship is upheld as one condition for patients and their families to attain meaningfulness, dignity, and a good death (Andershed, 2006; Franklin, Ternestedt, & Nordenfelt, 2006; Mok & Chiu, 2004).

While most research efforts have been placed on patients’ needs, feelings, and satisfaction with palliative care (Arnold, Artin, Griffith, Person, & Graham, 2006; Quintana et al., 2006), less attention has been given to the nurses’ feelings and experiences towards this kind of care (Boroujeni, Mohammadi, Oskouie, & Sandberg, 2008).

Additionally, nursing in hospital settings has been extensively studied compared to the homecare environment (MacDavitt, Chou, & Stone, 2007), and relational issues that look beyond the nurse–client dyad are rare (Lindahl, Lidèn, & Lindblad, 2011).

Current nursing literature on caring relationships tends to focus on the psychological attributes of the nurse (e.g., the ability to listen, being there) reflecting nursing ideology, which either helps or hinders the development of a trusting and respectful relationship (Browne, 2001). Tarlier (2004) indicates that such an individualistic approach may credit and/or blame nurses for “good” and “bad”—relationships, and little attention is given to personal or contextual elements (Doane & Varcoe, 2007).

To meet a patient in the home is to meet the inviolable domain of this person. This context is characterized by a high degree of patient autonomy, limited oversight of informal caregivers, and situational variables unique to each home (Ellenbecker, Samia, Cushman, & Alster, 2008). When the patient and the nurse meet in the patient's home, different expectations from both sides may govern the interaction. The nurse's role is that of a professional helper who is to meet the patient and show respect and dignity (Gäfvert & Ek, 1996). Maintaining a patient's dignity requires that the nurse get to know and understand the patient and, accordingly, carry out individualized treatment with respect to the patient's autonomy and integrity (Randers & Mattiasson, 2000). Studies have shown that nurses working in home care consider themselves guests in the patient's home (Öresland, Määttä, Norberg, Winther Jörgensen, & Lützén, 2008; Spiers, 2002). At the same time, they are also aware of their professional status, which occasionally dominates and, in turn, generates a demand to handle the question of distance and closeness in the nurse–patient relationship (Öresland et al., 2008).

Nurses and other health professionals have to be able to identify and articulate the nature of these interactions and their impact on patient outcomes, along with an understanding of factors that can promote or inhibit the therapeutic relationship (Weinberg, 2006). Therefore, to increase knowledge of the homecare setting, the aim of this study was to illuminate and interpret the meaning of nurse's lived experience when meeting severely ill patients’ expressions in the homecare setting.

## Method

### Procedure and setting

This study was conducted with 10 registered nurses (RNs) working in home nursing care in Mid-Norway. A purposive and convenience sample was employed to recruit nurses who had experience working with severely ill and palliative patients living at home. An invitation to participate was mailed with written information about the study, a consent form, and a stamped return envelope addressed to the first author.

### Participants and data collection

The participants consisted of six female RNs and four male RNs working in home nursing care. They ranged in age from 29 to 63 years (md=48). The RNs had worked between 4 and 36 years in health care as nurses (md=17.5). Data collection was carried out through recorded narrative interviews (*cf*. Mishler, 1986). The interviews were conducted with each RN in a preferred place chosen by the participants (either in their homes or in a meeting room at their workplace). The interviews lasted from 46 to 100 min. The interviewees were asked to narrate their lived experience of meetings with severely ill patients, in which their emotions had been particularly touched. The questions asked of the participants were: “Can you please tell me about a patient meeting in which you were positively touched?”, and: “Can you please tell me about a meeting, which awoke negative feelings?”. Questions were asked for clarification and to encourage exploration such as, “How did you feel? What happened? Could you please tell me more?”. The first author transcribed the interviews verbatim.

### Phenomenological-hermeneutic approach

The interview text was interpreted using a phenomenological-hermeneutic approach inspired by Lindseth and Norberg (2004). The aim of this approach was to reveal the meanings of lived experience through interpretation of the interview text. It is not the person narrating (the interviewee) the text that will be unveiled so much as the text message itself and the possible world the transcript reveals. The interpretation consists of a process comprising three phases, the naïve understanding, the structural analysis, and the comprehensive understanding. In the naïve understanding phase, the text is read several times with as open a mind as possible to achieve an initial understanding of the text as a whole. Structural analysis is an interpretative process, a detailed analysis, meaning unit by meaning unit, aimed at identifying parts and patterns of meaningful consistency to seek explanations of the text through outdistance and a critical way of being. The comprehensive understanding constitutes a dialectic movement between explanation and understanding; it is a way of seeing the whole in light of its part, and the parts in light of the whole. It is a critical, in-depth interpretation, taking into account the authors pre-understanding, the naïve understanding, and the findings from the structural analysis. Altogether, this interpretation produces a comprehension of what the text as a whole is talking about. The process of interpretation is not linear, but rather spiral, that is, a dialectic movement between the parts.

### Ethics

Participation was voluntary, and all nurses gave their informed consent and were guaranteed confidentiality. The participants were informed about the nature and purpose of the study and the intended use of research data. They were also informed about their right to interrupt their participation without giving a reason. The Norwegian Social Science Data Services granted permission for research (No. 34299), and it was carried out in accordance with the Declaration of Helsinki (World Medical Association, 2008).

## Findings

### Naïve understanding

Nurses meet a range of patient expressions during their home visits. These expressions leave different impressions on the nurse and do not depend on the individual patient's workload. Meetings that touch the nurses on a deeper level contain interactions that are dominated by either positive or negative feelings. Patient interactions, which influence nurses positively, mean sensing and recognizing the patient's vulnerability, and appealing to the nurse's need to be needed. Positive interactions entail satisfaction, pride, and competence within the nurse. Negative interactions, to the contrary, are characterized by violence and harassment that awake feelings of fear, anger, disappointment, and resignation. Sometimes, these emotions are not directly related to the patient himself, but are influenced by the presence of other people or the context surrounding the patient. Being touched by the patient's expression means an emotional engagement with another human being. This engagement represents a powerful tool, as it influences both the nursing activities towards the patient as well as one's self-evaluation of professionalism.

### Structural analysis

Several structural analyses resulted in four themes and seven subthemes illuminating the meanings of the nurses’ impression of the individual patient's expressions. In the presentation below all nurses are named “she” and all patients “he.” An overview of themes and subthemes is given in Table I.

**Table I:** Overview of themes and subthemes

Being open for the presence of the Other	Sensing vulnerability
	Empathizing with
Being satisfied	Feeling exceptional
	Being trusted
Being frustrated	Being disappointed
	Being angry
Being ambivalent	Being generous or reserved

### Being open for the presence of the Other

This theme reflects how expressions become evident within the nurse when she takes the patient and his home to heart. Entering a patient's home means experiencing the patients’ lives, sensing the milieu, and being surrounded by personal objects such as family photos and furniture, which all provide the possibility of absorbing a person's history and context. This environment personifies the patient somewhat, and both his strengths and weaknesses become more evident. The whole home setting seems to be the individual nurse's premise of her impression and feelings of sympathy for the patient and his relatives. Openness to the presence of the Other seems to presuppose an openness to the nurse's own existence and emotions.

#### Sensing vulnerability

Letting the patient's expression make an impression means facing humanity. Becoming aware of and recognizing the vulnerability in the patient's situation highlights these interactions. Sometimes, these connections appear in settings where the patient's vulnerability is obvious, for example, in dying patients. One nurse said

When a patient knows he is going to die … it's something with his eyes … they are totally abyssal …. *It's the helplessness that afflicts me. I want to help*.

It is not only the eyes that give impressions; the patient's appearance is also significant. If the patient is viewed as a kind and gentle person the nurses’ willingness to care seems to grow; it confirms feelings of commitment. One nurse said

… in a way he evokes care … a will to give good care. He gives only positive back. He's so grateful and I'm glad I can help him in large and small … // … he touches me ….

At other times, sensing vulnerability is facilitated by the nurse's ability to identify with the situation or if she has the time to build a relationship with the patient. One nurse said

He was interesting as a person as well … he had something that reminded me of my own grandfather. Maybe that was important. He had this courage.

The relatives’ vulnerability is also a centre of attention. Different aspects of losses and changed relationships engrave their situation and appeal strongly to the nurse's compassion. Sometimes, the illness can change the patient's behaviour, and he might become physically and verbally aggressive towards other people. One nurse said

I remember a patient with a brain tumour who lived at home with his family. His wife had thought ostensibly that everything would go well at home. Instead it became totally changed. He became aggressive and violent and difficult to handle for his wife …

On such occasions, the nurse becomes a supporter to the family. The nurses feel that the relatives appreciate and are comforted by their care but they also sense that relatives are anxious about their becoming sick. Involving and trying to understand what the family is living through provokes a self-reflection within the nurse and she becomes more conscientious of her own values and attitudes towards life. This process reveals a mutual sensitivity; a shared vulnerability among the nurse and the patient and his relative. Naturally, facing death and dying is a reminder of human fragility and patients may have a need to talk about what will happen to them when they die. Approaching such questions and trying to comfort, often means falling short and raises feelings of powerlessness. One nurse said

I feel so insufficient … that's the worst. I am not so good with words. And what is there to say? Nothing is going to help. It won't get any better. In the end, when we are dying, we all have to go the way by ourselves …

#### Empathizing with

Meeting younger or severely ill patients touches the nurses significantly and implies a strengthened ability to participate in the feelings of the Other. Even if such situations make them insecure in their work, they recognize a total dependency. This dependence awakes a responsibility within the nurse, which is not strictly related to healthcare, but to other activities in daily life as well. For example, looking after a patient's appearance and combing his hair, that little extra, becomes an important duty. One nurse said

It's tough enough to meet a mother that cannot take care of her children because of her illness. It's easy to imagine, to acquaint with …. I know how I would like to have it; how my appearance should look like if I was in her situation …. I would like to look good …. It's about respect.

Empathizing with means to sense wholeness in the patients’ situations. Impressions of their housings, relationships with their family and their life stories are melted in the nurse's imagination and affect her sensitivity to the “here and now” situation. Sometimes, the nurse finds herself overwhelmed. One nurse said

His whole life-situation was so tragic … chained to this wheelchair … and his wife seemed so cold. He was a man of few words … but his eyes … it made me wonder: What was the content in his life?

The length of contact with the patient does not always seem to matter. One nurse told about a short, nightly visit to a patient whom she visited just once:

… and then I was in the hall and saw all these small shoes …. It was sorrowful. I remember I thought … soon there's no father in the house. It was sorrowful.

#### Being satisfied

Patients’ and relatives’ expression often mean satisfaction and contentment for the nurse. The contentment reaches beyond what can be seen as a conjunction of providing good practical care or being a good caregiver. It affects her self-esteem and confirms her value as both a fellow human being and a professional nurse.

#### Feeling exceptional

As opposed to working in institutional care, home nurses find themselves treated as visiting strangers and generally warmly welcomed into the patient's home. Being invited into the patient's home and life is seen as a gesture, which becomes even more meaningful if the patient wants to share his existential worries. To have the privilege of talking about essential issues gives a sense of pride and of having a special status. Moreover, this feeling grows when patients distinctly entrust all power in the nurses’ care. One nurse said:

He told me this sad story, and blamed himself for being selfish and greedy … and this …. All these years he had told nobody about it, but now … he told me, and we sat there, and we both cried.

When it comes to delivering care, availability of professional support or geographical challenges is other dimensions, which differ in the homecare setting. Home nurses may feel called upon to face nearly-impossible situations. For example, they may be forced to use a snowmobile to get to the patients when all of the roads are impassable in winter. One nurse said

It's important that the patient is well at home, but no one asks how it's done, - we must solve it ourselves.

At other times, the nurse finds herself selected by her peers. In extreme situations, it may be that colleagues avoid visiting the patient. Such situations could be the result of the nurse being subjected to extreme living conditions where hygiene is somewhat lacking. To be professional at these times is not always experienced as easy. Being the one who manages gives strength. One nurse said

When we opened the door you had to push aside cat excrement. He had a lot of cats. It was so … it smelled terrible, and I remember I thought, - Can they really let a man live like this? It was terrible living conditions. We didn't do anything about it. He was in the terminal phase and wanted to die at home. I remember I thought he was nice and didn't mind visiting him. There were many who didn't want to go there for the smell of the so … the smell of the clothes when you've been there …. I didn't mind going there. I managed what few of us did.

#### Being trusted

Mutual trust and understanding between the nurse and her patient is highlighted in all of the narratives. A sense of wellbeing and feelings of safety and confidence arise within the nurse when patients gain from, and are satisfied with the help they receive. Nurses characterize sincerity, openness, and flexibility as elements of good communication and care. At other times, nurses stress the importance of having the same private interests as the patient:

We had the same interests. I was the only nurse that was interested in hunting. We got a very close contact. One day, before he died, he was trans-located to the hospital without my knowledge. When I heard that, I visited him daily on my free time.

The narrations of caring for severely ill patients and palliative patients reflect personal bounds between the nurse and her patient. Sometimes, the nurse has to master complicated procedures or medical equipment. On those occasions, her feelings of competence seem to rely more on her “being” than her “doing.” One nurse said

I remember a patient, an old lady with cancer who was dying. I sat down a while at her bedside. She (the patient) said ‘You must not leave me’ …. I can still hear those words. I promised I'd stay and hold her hand … it seemed as she became calm and safe because of it.

#### Being frustrated

The theme being frustrated expresses the nurses’ feelings of disappointment, anger, and grief. Working in home nursing care means being in rapid succession with other people, and sometimes being exposed to negative or non-understanding expressions.

#### Being disappointed

Nurses are sometimes exposed to aggressive patients, for example, patients with progressive dementia. Such occasions can be difficult to handle, and the nurses have to rely on their creativity and ability to establish an alliance with the patient's relatives. Sometimes, nurses meet relatives who are controlling and distrustful, and who constantly undermine their strategy towards the patient. One nurse told about her experiences from one such episode:

Relatives were very distrustful and doubted my competence. Sometimes I felt very subservient when I met them. It was as they looked down on me. They always let me down when I met them.

Feeling humiliated by the relatives may affect the nurses’ actions negatively. After episodes of humiliations, they explained how they agree with the consequence that the patient does not get as much help, time, and support as he needs. In these instances, the nurses evaluate their care to be shortened, though reasonable.

At other times, the nurses feel as if the patients bother them or are fractious; this also causes feelings of disappointment. According to the informants, this is often connected to self-damaging actions and aggressive or other disruptive behaviours. One of them said

I try my best and he treats me in this way, so aggressively …

These actions draw out negative sides in the nurse and sometimes result in negative speech. At other times colleagues do not believe or support the nurse when she tells about her experience when visiting the patient's home. One said

When I told my colleagues about my feelings, they didn't understand …. I had no back-up from them.

#### Being angry

Feelings of anger emerge when meeting demanding patients; patients who try to dominate the nurse or their family. Sometimes nurses encounter situations with domestic violence. One said

He was very aggressive especially towards his wife. // She talked with me and suddenly he became verbally furious. When this happens you feel that you get palpitations …

Furthermore, emotions of anger and unfairness are expressed regarding the suffering that the patient and his family have to endure. Self-expectations are high, and when the nurses feel that they are not being “strong enough” to have the total focus on the patient, they become annoyed with themselves. Awareness of how personal coping strategies influence and motivate their caring actions can be distressful. One nurse talked about this,

It happens when you enter a home … and you just know how hard it's going to be …. For example, this patient has refused moving to a nursing home, but you can see how worn out he is … and his relatives are also exhausted. And you can envision that your own work situation will be hard. In these situations I must admit that sometimes, I have “pushed forward” resettlement to a nursing home. I am afraid, that's the way it is … sometimes my own needs come first.

#### Being ambivalent

Throughout the patient's illness trajectory, coexistence of contrasting and sometimes conflicting feelings are illuminated in the nurses. These feelings are perceived as distressful and at times described by the nurses as an emotional, roller coaster. Following and responding to these feelings have consequences on the care they provide.

#### Being generous or reserved

Sometimes the nurse becomes so touched by the patient that she emotionally “takes him home” after work. One nurse explained,

I was touched somehow … He had this charisma, which caught me, and made me accompany him …. Perhaps, I became less professional, but I felt I should be there for him and help him in the best possible way …. He was always on my mind.

On such occasions nurses express a need to have a decorous farewell, and if they miss it, they have a bad conscience. One said

We're not robots …. We have emotions too.

At other times, the nurse may feel exposed to close scrutiny and find the patient's demands difficult or impossible to handle. Even if the patient's vulnerability and needs are obvious to the nurse, patient's demands may be of such nature that the nurse does not feel good in the situation or she may feel totally controlled. One nurse said

I feel that I have burned myself out on some patients … You feel in a way empty and that you cannot give any more extra … // … some patients give all those tasks. Mere trifles, I think … for example, how you should make the bed with all the pillows and blankets at the right places. In the end you get so tired.

The coexistence of negative and positive emotions influences how the nurses perform their caring actions. Pushed to extremes: to be positively touched is followed by being inviting and striving to do the little extras. However, being touched negatively may lead to resignation or withdrawal. Neither being generous nor being reserved seems preferable. Both approaches have their extremes and these borders are blurred when it comes to professionalism. One nurse talked about a meeting with a dying patient,

But, this I have learned; it is not about me; it is about the patient and his relatives. I became overinvolved with this man …. I have thought much about this - I took too much space. I was not so professional …

Another nurse said

Some patients require something all the time, without having the largest caring needs, as I see it. Then I am not so good at giving that little extra … I become more distant, and I have to work more with myself to be a good nurse.

Though the nurses’ morals guide them to treat all patients equally, their stories reveal a different reality. Without judging their actions as clearly unethical, they still find it imperative to explain or even defend the fact that patients are receiving different care. Balancing this power of emotions means mobilizing feelings in some situations and suppressing them in others.

## Comprehensive understanding

Patients’ expressions that make impressions in nurses create emotional waves. Expressions leave impressions that call upon the nurse, and confront her with taking the risk of letting intuition and pre-reflexive feelings enter into her caregiving. Allowing for the Other's presence is seen as a precondition, which means facing humanity and sensing vulnerability in herself as well as in the Other. Understanding and balancing this emotional dimension in care seems to cause confusion and distress within the nurses. Realizing how their feelings may lead to either generosity or aloofness towards the patient is upsetting. Our interpretation suggests that these impressions echo confusion according to the role of being a professional nurse.

## Discussion

The aim of this study was to illuminate the meaning of the impressions of patients’ expressions, when nurses meet severely ill patients living at home. We found four themes, further broken down into subthemes that shed light on the interviewee's experiences: “being open for the presence of the Other,” “being satisfied,” “being frustrated” and “being ambivalent.” We found that expressions left impressions that caused emotional waves within the nurses. The wakening of emotions influenced their caring actions, leading to an imbalance according to ethical preferences as well as their self-esteem as professional nurses.

Working in a home environment facilitates the nurses’ opportunities to grasp a more comprehensive picture than what might be possible in a hospital ward. The home can be metaphorically viewed as a display window of the person's identity (Lantz, 2007). A person's history, life context, and identity is assimilated and established in his home, which makes it personal, private, and individual. Caring at home may be looked upon as a shaped professional friendship (Lindahl et al., 2011) and some even state that the friendship dimension should be a part of the professional relationship (McCann & Baker, 2001). However, this friendship is a balancing act, because power is an underlying issue. Nurses are empowered by their expertise and their mission, but disempowered because they have to adjust to the patient's home. Patients are empowered by being at home, but disempowered by their dependency on care (Spiers, 2002). Accordingly, this friendship differs from other kinds of friendships because those involved have not chosen each other voluntarily.

When caring for a patient who emotionally touches them, the nurses revealed that they were exposed to an emotive situation where they opened themselves to the patients’ and relatives’ vulnerability. The nurses in this study felt sympathy towards the patients and were emotionally involved through the physical milieu they visited, that is, the patient's home. “Being open for the presence of the Other” means that nurse understand the patient and his surroundings through their senses. According to Løgstrup (1987, pp. 87–94), this sensory sensibility is important for human's wellbeing. Also important is being responsive and having a sentience of understanding of other people's subjective situations that affect the relation to oneself, that is, an understanding of oneself in light of other people (Gadamer, 1994). This means that, working in home nursing care and meeting people in vulnerable situations, often suffering people at the end of life, the individual nurse meets not only the patient and his relatives; she also meets herself (Chen, Del Ben, Fortson, & Lewis, 2006).

Nurses may relate to the patient and his situation in a distanced, reflective way or in an emotionally engaged, immediate way. In the latter, the understanding of the experience, just as the feelings that it provokes seems to be both spontaneous and inseparable. According to Løgstrup (1995), there is an indissoluble uniting between understanding and impression. In our study it seems as the impression of the patient's and relative's situations are built on an inter-human relation where individuals confirm each other (*cf*. Buber, 1966). This means that nurses in the situation see, hear, and respect the patients’ will and through her actions confirm his dignity (Kalfoss, 2010).

“Being satisfied” refers to the important fact that the patients confirmed the nurses in our study. Closeness and intimacy are natural elements in the care for severely ill and palliative patients in home nursing care, and it demands emotional engagement from the individual nurse (Hamilton & McDowell, 2004). Sometimes, the presence does not need words; it is sometimes about a silent kinship and creating something together without any noticeable activity. Confirming the patients and establishing a calm and secure atmosphere is essential in home nursing care (Hamilton & McDowell, 2004; Pavlish & Ceronsky, 2009). In our narratives, it becomes clear that this confirmation goes both ways—patients and relatives also confirm the nurse. According to Irvin (2000) and Pavlish and Ceronsky (2009) among others, the relationship is characterized by an engagement that passes on to a more social mould where the parties are close to each other in a way that is not common in traditional nursing care, a relationship which is more familiar than professional to its character (Barnard, Hollingum, & Hartfield, 2006). Sandgren, Thulesius, Fridlund, and Petersson (2006) emphasize that it is necessary for the nurse to be personal in the relation with the severely ill or palliative patient, personal without being private. However, there is an obvious risk that nurses in home nursing care may lose their objectivity and become overinvolved in the patient who confirms. To the contrary, they may also run the risk of becoming inattentive to the patient if the focus is on more medical involvement or on their private needs. The latter may allude to the need of being a “clever nurse” or being indispensable. If that happens, it seems to be a danger that the relationship could change from a dialogue to a monologue.

Working in home nursing care also means the possibility of exposure to negative and non-understanding expressions. In these instances, stressful feelings may arise—*feelings of frustration*. At times, patients or family members may be perceived as unpleasant or excessively demanding. The nurses’ feelings of unfairness or resignation seemed to be of significance when they explained instances in which their caring actions were shortened. Nurses do not treat all patients and relatives equally. They have patients they like and patients they dislike or experience as onerous (*cf*. Hellzén & Asplund, 2006; Lilja, Hellzèn, & Hellzèn, 2006). The nurses talked quite freely and honestly about meetings, which brought about this kind of negative emotions. However, when reflecting about providing less care to these patients, the nurses indicated feelings of inadequacy. The meaning of caring for a patient who exhibits negative and non-understandable actions seems to touch upon moral aspects of the human existence. Our material suggests a complexity in how the nurses subjectively experience these negative expressions in which we have only scratched the surface. In the area of psychiatric and mental health nursing, there are studies focusing on nurse–patient interactions, where the patients have specific disorders (e.g., borderline; Woollaston & Hixenbaugh, 2008, or dementia; Robinson & Cubit, 2007), but we have not found any empirical study from the general homecare setting.

The theme “being ambivalent” refers to the nurses’ experience of contrasting emotional commitment towards the patients’ expressions. Meeting a patient's expression may work as a reminder of their own humanity and frailty, and the consciousness about “personal luggage” may be stronger. Experiencing this may entail both anxiety and content, which naturally may influence how the nurses view themselves as professional nurses. A positive self-image and professional identity are seen as preconditions for nurses in order to have a strong and therapeutic relationship with a patient (Schütz & Tice, 1997). The nurses in our study often excused themselves for not being professional enough when they addressed their personal engagement. It seemed, somehow, that the strong emotional involvement (both positive and negative in nature) conflicted with their ideal of being a professional nurse. Blomberg and Sahlberg-Blom (2007) found that nurses balanced between being close and distancing themselves when confronted with severely ill patients. In most situations, the strategy seemed spontaneous, but it could also be a more conscientious approach trying to protect oneself from painful feelings. Interestingly, the nurses in our study did not address concerns about being burned out. Rather, their worry seemed to concentrate on whether “feeling too much” could affect the adequacy of care. Fisher (1995) found that nurses who approached patients in a humanistic and personalized manner experienced better relationships and provided better care than those who adapted a manner consistent with a traditional medical model. On the contrary, by acting in a humanistic person-to-person manner, the nurses in Fischer's study scarified an authority needed for medical effectiveness. Apesoa-Varano (2007) raises the question as to whether professionalism is compatible with caring. Caring as a concept is problematic to define, and educators still lack consensus on what caring actually is. Davies (1995) argues that emotions, affections, and relationships are core elements of care, and also asserts that caring has been ignored or seen as unskilled work, and has thus remained under-theorized and inadequately conceptualized. Whatever personal attributes or best intentions to care the individual nurse may have can be altered to reflect those of the profession to which they belong. If the inherent professional view of caring is highly idealistic or not explicitly defined, it may place an almost-impossible burden of responsibility on the nurse. We may have identified this tension between a scientific ethos within professionalism and the mothering ethos of caring (Apesoa-Varano, 2007). However, our study does not allow for any conclusions other than pointing out the importance of further research in this area.

The findings in our study can be understood from Buber (1966) who states that every human being requires significance —, that is, being placed in another's world. Our findings indicate that one meaning of meeting the individual patient's expressions is that the nurse and the patient occasionally participate in one another's lives, not purely psychologically. According to Buber (1966) meeting the other's expressions also ontologically as a manifest reality of the between, that is, as the inter-human. In such occasions each one of the actors turns to each other in order to communicate person-to-person.

Communicating person-to-person may be the art of nursing, as it is found to be featured by the nurse's mode of being, knowing, and responding (Gramling, 2004). In the referred study, the art of nursing was found to be an attunement rather than an activity.

Professional competence comprises a sufficient level of knowledge and skills to provide effective care (Pope, 2012). McCance, Slater, and McCormack (2009) explain that nurses may lack confidence in their abilities, appear untrustworthy from a patient's perspective, may lack the ability to prioritize workloads, and be unable to understand patients as individuals. Lack of competence, as well as poor communication skills and an unstable nurse–patient relationship, may have a detrimental effect on patients’ physical and psychological health.

The nurse–patient interaction seems to profit by the nurse being obliging and willing to spend time on getting to know the patient through his own stories. In other words, it seems important for the nurse to invite the patient into a “room of awareness,” a room where he will be heard and accordingly also hear himself. This attitude is similar to the phenomenological attitude *epoche*, where we reveal the phenomenon's essential meaning through putting our given perceptions and opinions of the phenomenon within brackets (*cf*. Fink, 1995).

If the nurse is willing to let the patient's expression make an impression on her without hiding behind the protection of foreknowledge in the initial meeting with the patient, he will feel invited and his narration will help to orientate him in life (*cf*. Fink, 1995; Lindseth, 2012). However, there is a risk that the nurse only reflects out from what she already knows, that is, her professional knowledge. If the nurse meets the patient with her prior knowledge and a preparedness to categorize what has been said, explanations and models will appear, and the “room of awareness” will be locked. In that case, the patient's expressions will be reduced to mere information, and the nurse may or may not choose to act on it. Based on our interpretation, we believe that the confusion according to the professional role should be taken most seriously. What these nurses have communicated mirrors a need to reflect over daily care and to become aware of what governs their caring actions.

## Methodological considerations

It is possible to interpret text in different ways. Our findings constitute one of several possible interpretations (Ricoeur, 1976). The phenomenological perspective focuses on the person's lifeworld and lived experience, and thus requires openness to the interviewee's experiences (Lindseth & Norberg, 2004). The gateway to this lifeworld goes through the narrative, with all its symbols and metaphors, which have to be interpreted by using both explanation and understanding (Ricoeur, 1976). In line with the hermeneutic tradition, issues of trustworthiness are a matter of rigour throughout the process which aims to arrive at possible interpretations—not finding the absolute truth. The first author carried out the initial analysis; however, all three authors have reflected on and continuously and critically worked with the assessments until a consensus was reached. Our interpretation represents what we have found to be the most useful way of understanding this phenomenon. Therefore, this research offers one perspective, which may constitute a basis for further reflections about when the patient's expressions become the nurse's impression, that is, when the nurse is emotionally touched by the patient's expressions.

## Conclusions and implications for practice

Discussions and reflections about how the patient's expressions make impressions in nurses in home nursing care are highly desirable to evidence-based practices, and to improve care in relation to ethics and the staffs’ professional identity development. Confident and trusting relationships based on the nurse's ability to reconcile humanity and professionalism is of utmost importance to severely ill or palliative patients. In this way, patients may experience sufficient support. When the patient's expression makes an impression on the RN, a “room of awareness” will open where it is possible for the patient to appear in a greater clearness. This will enable the nurse to hear and understand the patient in a more conscious way. The nurse will then be able to encourage the patient's ability to find new orientations in what he communicates and allow for the possibility of reconciling with the illness and the present situation. There is a need to pay more attention to how the emotional dimension in caring is understood and impacts the way nurses perform their professional role.
